# Statistical and visual differentiation of subcellular imaging

**DOI:** 10.1186/1471-2105-10-94

**Published:** 2009-03-22

**Authors:** Nicholas A Hamilton, Jack TH Wang, Markus C Kerr, Rohan D Teasdale

**Affiliations:** 1 Institute for Molecular Bioscience, The University of Queensland, Brisbane, Australia; 2 ARC Centre of Excellence in Bioinformatics, The University of Queensland, Brisbane, Australia

## Abstract

**Background:**

Automated microscopy technologies have led to a rapid growth in imaging data on a scale comparable to that of the genomic revolution. High throughput screens are now being performed to determine the localisation of all of proteins in a proteome. Closer to the bench, large image sets of proteins in treated and untreated cells are being captured on a daily basis to determine function and interactions. Hence there is a need for new methodologies and protocols to test for difference in subcellular imaging both to remove bias and enable throughput. Here we introduce a novel method of statistical testing, and supporting software, to give a rigorous test for difference in imaging. We also outline the key questions and steps in establishing an analysis pipeline.

**Results:**

The methodology is tested on a high throughput set of images of 10 subcellular localisations, and it is shown that the localisations may be distinguished to a statistically significant degree with as few as 12 images of each. Further, subtle changes in a protein's distribution between nocodazole treated and control experiments are shown to be detectable. The effect of outlier images is also examined and it is shown that while the significance of the test may be reduced by outliers this may be compensated for by utilising more images. Finally, the test is compared to previous work and shown to be more sensitive in detecting difference. The methodology has been implemented within the iCluster system for visualising and clustering bio-image sets.

**Conclusion:**

The aim here is to establish a methodology and protocol for testing for difference in subcellular imaging, and to provide tools to do so. While iCluster is applicable to moderate (<1000) size image sets, the statistical test is simple to implement and will readily be adapted to high throughput pipelines to provide more sensitive discrimination of difference.

## Background

With applications such as drug discovery [1], the ability and the desire to experimentally determine protein localization and trafficking is leading to a rapid growth in cell image data sets in need of analysis on a scale comparable to that of the genomic revolution [2,3]. A key problem in location proteomics is that the analysis and comparison of localizations is largely performed by the slow, coarse-grained and biased process of manual inspection. Just as algorithms such as BLAST have been developed to search, compare, cluster and draw conclusions from the sequence information of the genome revolution, it is critical that a similar suite of tools be developed for the flood of bio-imaging to maximise its benefit.

Towards this goal, image statistics have proved invaluable in the analysis of fluorescent subcellular imaging. Measures of features such as texture and morphology (for instance, the length of the perimeter of the object of interest) in combination with machine learning methods such as neural networks and support vector machines have proved highly successful at classifying subcellular images of the major organelles of a cell, and have achieved near perfect accuracy [4-6]. However, a difficulty with such systems is that organelle structure can vary widely between each cell type, and thus automated classification systems usually require that they be re-trained for each cell type, though research is ongoing in removing this limitation [7]. Another difficulty is that subcellular localisation classes and representative training images for each need to be chosen prior to training. Hence automated classification is to some extent fitting an image into a pre-defined box which may not necessarily reflect the true diversity of protein expression within the cell. Despite these limitations, the question of "where is the protein in the cell?" can readily be answered using automated classification, and these techniques have been applied to the whole yeast proteome imaging [8] and demonstrated that automated classification can produce high confidence classifications on real world high throughput imaging [9].

**Figure 1:**
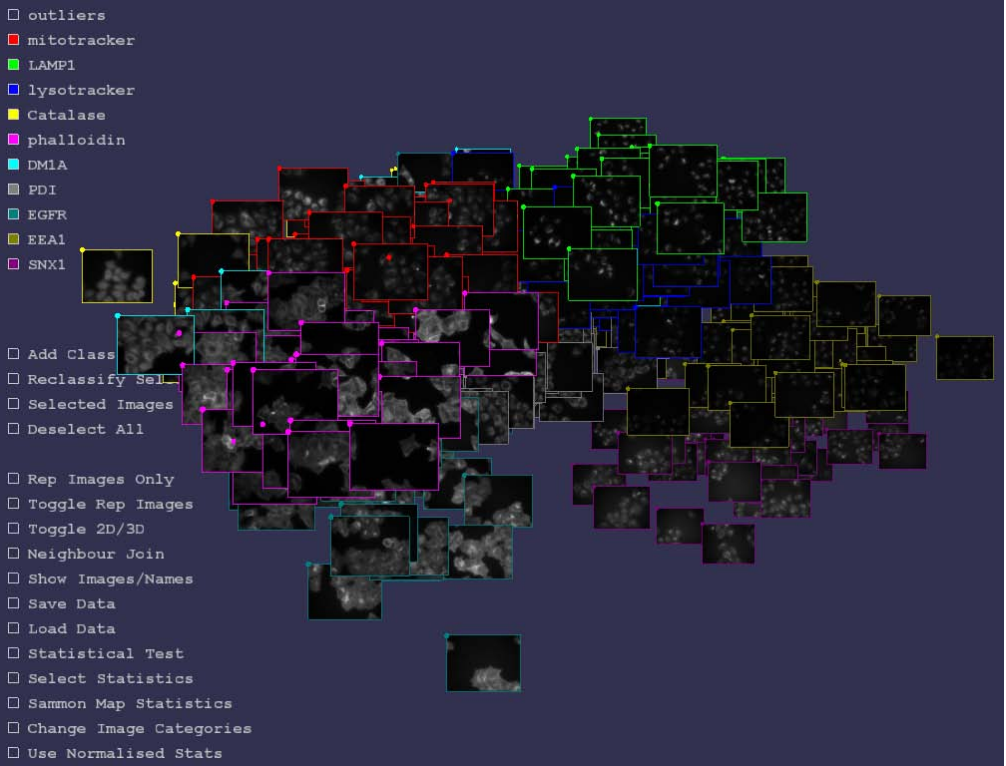
**The 500 images of 10 fluorescently imaged protein subcellular localisations of Image Set A visualised in iCluster**. Each border color represents a different sub-cellular localization. The images are automatically spatially placed in 2D or 3D such that the statistically similar images are close to one another. The spatial placement algorithm only uses the statistics, and is not aware of the subcellular localization categories, these are only used for border coloring. Note the strong clustering of each subcellular localization class, showing that the statistics and algorithm can readily distinguish the localization images. The user may browse, navigate and interact with the image set, show/hide images, show representative images for each class, select subsets of images, detect outliers, reclassify images, and perform tests to give p-values for whether two images classes are different (for instance comparing treated/untreated cells).

Here we describe a methodology, protocol and software for testing for differencein protein subcellular fluorescent imaging. It draws together a number of components into a single framework, called iCluster, for the visual and statistical differentiation of bio-imaging. The first component is threshold adjacency statistics (TAS), a type of image statistic specifically designed to distinguish subcellular imaging to a high degree while being fast to calculate [5]. TAS are then utilised for statistical testing and visualisation. In the visualisation component, TAS are Sammon mapped [10] into 2 or 3 dimensions in such a way as to preserve the distance relationships between image statistics vectors, and the images are visualized at the coordinates so determined in 2 or 3 dimensions. An error term gives feedback on how well the distances have been preserved by the mapping and is defined in detail in [11]. The effect is that those images that are statistically similar are spatially close, thus enabling patterns of difference and similarity to be readily recognized in large image sets. The user can also navigate through an image set, visualize different classes of images for comparison, show or hide classes of images, select and reclassify images, show a representative image [12,13] for each class of images, and export data or create images of the results. This enables the distinct patterns of protein localisation or distribution across the image set to be readily seen, while also allowing images to be viewed at high resolution. In this manner outlier images may be found and either removed from further analysis, or reviewed in detail if it appears that they form a subclass of protein expression in their own right. Further, when comparing treated and untreated experimental imaging, changes in localisation may be observed as the treated/untreated images forming distinct clusters. Figure 1 shows a snapshot image taken of visualization in iCluster [see Additional file 1 for a movie demonstration]. An early prototype of iCluster showing the core principle of spatial layout by statistical similarity was described in [11].

The aims of the current work are three-fold. Firstly, we introduce a novel method of statistical testing, the *centroid distance test*, for comparing image sets and returning p-values for the null hypothesis, that is, there is no change. Comparison to previous tests shows the centroid distance test to be significantly more sensitive in detecting difference in subcellular imaging. While the work we describe here has been implemented in iCluster, the statistical test is simple to implement and hence could readily be applied within other image analysis pipelines. Secondly, by examining the core issues in establishing an image analysis pipeline such as "How many images are needed?", "Do cells need to be selected from the images?", "What is the effect of outliers?" and "How subtle an effect can be detected", the aim is to outline a protocol for creating a workflow. Finally, by releasing the iCluster software the hope is that there will be a much wider uptake of quantitative methods within the bio-imaging community to truly enable the many benefits that the new high throughput microscope technologies offer.

iCluster is being released with this publication and is available for download under the GNU General Public Licence from . It is available for Windows, GNU Linux and MacOSX and includes source code. A java applet demonstration is also available on the same site.

## Results and discussion

**Figure 2:**
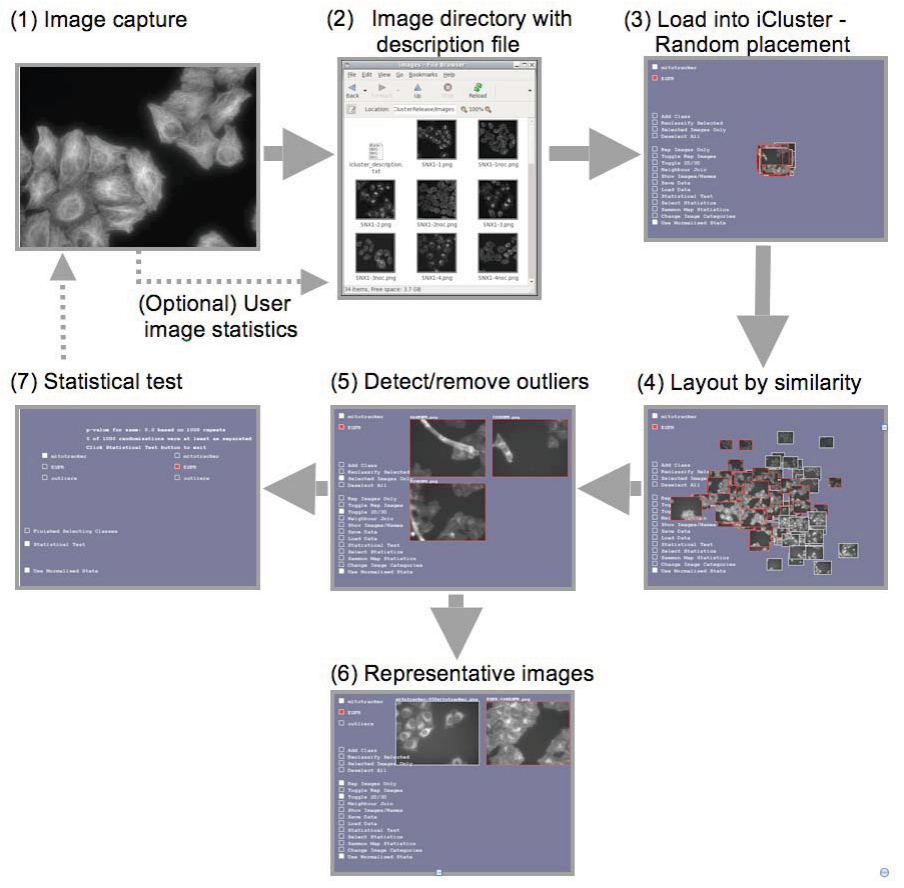
**iCluster workflow to test for difference in imaging**. (1) Treated/untreated sets of images are captured using identical microscope settings. (2) Images are stored in a directory together with a simple text format file describing which experiment each image belongs to. (3) The image description file together with the images is loaded into iCluster via a file selector. If (optional) image statistics have not been supplied by the user in the description file, TAS are generated automatically. Initially images are randomly placed in 3D. (4) The user initiates spatial layout by statistical similarity by clicking on 'Sammon Map'. (5) Once layout has finished, outlier images are found, viewed in detail, reclassified as 'Outliers', and removed from view. (6) Representative images for each experiment are automatically found and viewed in detail. (7) p-values are then calculated for the null hypothesis: no shift in localisation has occurred. For experiments where a large number of treatments are imaged, an initial test run on one or more treatments might be used to determine a minimum number of images required to detect difference. See also Supplementary Movie.

A key requirement of many imaging experiments is to determine whether there has been a change such as a shift from one subcellular localisation to another or a re-distribution within the cell of the organelle containing the protein. Typical experiments would be to image a protein with and without co-expression of another protein in order to understand how they interact [14], or to image a protein under a range of drug treatments to screen for active compounds [1]. In such cases it is not so important what the actual localisation of the protein is so much as whether it has been perturbed by an introduced interaction. In the following, the core issues in establishing a workflow for testing for difference are considered with examples given for image sets A and B (described in Methods). A summary of the workflow is given in Figure 2. The aim is to test the limits of the centroid distance test (see Methods) and establish a protocol for application to other image sets.

### To crop or not to crop

Depending on the application it may be beneficial to calculate image statistics for individually selected cells. For a screen in which cells are relatively uniform across the population, selection might not be required, while for transfection experiments in which cell populations may be more heterogeneous selection may be recommended. Avoiding cell selection can be advantageous in that automated selection methods can give variable results, especially when cells are confluent on the slide. Selection will typically involve experimenting with a variety of softwares to find the one that best suits the assay.

One of the advantages of threshold adjacency statistics (TAS) (see Methods) is that they may be calculated either for images containing multiple cells or for images in which individual cells have been selected. In [5] it was noted that classification accuracies using support vector machines with TAS on multiple cells per image or selected cells were comparable. Hence images may be pre-processed before input to iCluster using dedicated cell selection software to give individual cells, or raw images containing multiple cells may be directly utilised.

To avoid confounding results by variability in the success of cell selection, here we test on images for which no pre-processing for selection or cropping has occurred.

### Detecting outlier images

**Figure 3:**
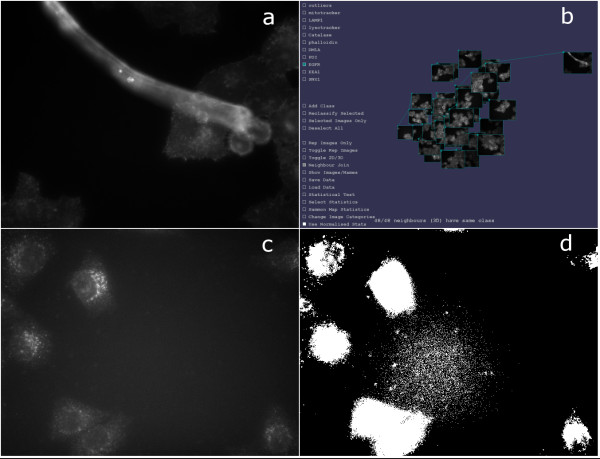
**Outlier images**. Images that are statistical outliers may be caused by a protein localisation that is distinct from the majority of cells imaged; artefacts in imaging; or an artefact in the generation of the statistics. (a) shows an imaging artefact, perhaps caused contamination of the slide. (b) shows the same image (upper right) in the context of the 3D placement by iCluster of other images of the same class. (c) shows another outlier image found using iCluster. In this case a non-uniform background has caused the automatically generated region of interest selection mask (d) to select non-cellular regions, thus skewing the statistics calculation.

For each of the 10 classes of image from set A, outliers were detected by viewing that class of images within iCluster and observing which images did not cluster with the main group. Other approaches to outlier detection include removing those images at greater than 3 standard deviations distant from the mean [15]. A total of 17 images that were spatially distant from other members of their class were found, with between 0 and 3 outlier images per class. Closer examination of the outliers showed each to be the result of either an imaging artefact or a poor selection of cellular regions (Figure 3). In the following, analyses will be performed on Image Set A both with and without outliers in order to gauge their effect on the statistical analyses.

### Image number

A key question in automated image analysis is how many images are required to achieve statistical significance in detecting difference. Towards this, p-values for the null hypothesis for all pairs of the 10 image classes were generated as follows. For a given pair and an integer n, a random subset of n images of each was selected, and the p-value for the null hypothesis calculated. This was repeated 20 times for that pair (with different random subsets) and integer n. Hence for each pair of classes and integer n, 20 p-values were recorded. For each n, the worst (highest) p-value over all the pairs and the 20 repeats then gives an indication of how well a set of n images of two distinct localisations may be distinguished. The results of this process are given in Table 1 for Image Set A, both with and without outlier images.

**Table 1:** Worst case p-values for subsets of Image set A

n (# images)	5	6	7	8	9	10	11	12	13	14	15	16	17	18	19	20
Worst p-value	.665	.541	.428	.611	.293	.249	.144	.245	.128	.103	.077	.045	.034	.055	.047	.017

Worst p-value (no outliers)	.337	.267	.134	.076	.117	.045	.080	.043	.029	.023	.012	.008	.007	.006	.005	.002

For each pair of the 10 classes of Image Set A, a subset of *n *images was randomly selected from each, and a p-value calculated for the null hypothesis of no difference. This was repeated 20 times for each pair. For a given n, the highest p-value over all pairs and their 20 repeats is shown in the table. P-values are shown for the cases of removing or not removing outlier images before random selection.

It can be seen that the inclusion of outlier images significantly increases the p-value for a given image set size, hence reducing the confidence with which the null hypothesis may be rejected. To achieve a 95% confidence level (p-value < 0.05) requires 19 images with outliers included, while only 12 images are needed when outliers have been removed. Hence outlier removal while not essential if their number is relatively small, greatly improves confidence.

Two classes of image from Image Set A that are visually and statistically similar are plasma membrane (EGFR) and actin cytoskeleton (phalloidin). To gain an understanding of how well these might be distinguished for different numbers of images, a similar process to the above was tested on just this pair. Random subsets of n images were generated, and p-values calculated. For each n this was repeated 10 times and the average p-value over those 10 was recorded, the results of which are given in Table 2.

**Table 2:** Average p-values comparing images of plasma membrane and actin cytoskeleton images

n (# images)	5	6	7	8	9	10	11	12	13	14	15	16	17	18	19	20
Average p-val	.083	.072	.088	.054	.042	.025	.033	.015	.003	.011	.015	.006	.010	.004	.003	.001

Average p-val (no outliers)	.094	.116	.032	.032	.017	.017	.014	.004	.005	.008	.002	.002	.002	.001	.001	.001

For a given number *n *of images, random subsets of *n *plasma membrane and *n *actin cytoskeleton images were selected and a p-value calculated. For a given *n*, the average of 10 repeats is shown for the cases of removing or not removing outliers prior to selection.

Again, it can be seen that outliers degrade confidence in rejecting the null hypothesis, though once 9 or more images are used both cases (on average) achieve 95% confidence. Overall the results of Tables 1 and 2 suggest that outlier removal is to be recommended and that a reasonable number of images to collect to differentiate image sets of these types would be 20, allowing that outlier removal might leave 15.

### Detectability

Two issues may arise in using image statistics to detect difference in imaging. The first potential problem is in whether the statistics are able to detect relatively subtle but discernable differences. The second is whether the statistics are overly discriminating, that is difference is detected when there is none or little, perhaps due to changes in imaging conditions rather than due to a redistribution of a protein within the cells. When testing for changes in a protein's subcellular localisation under treatment, over-sensitivity may be controlled by ensuring that microscope settings such as exposure time and imaging conditions are identical for all image sets compared.

**Figure 4:**
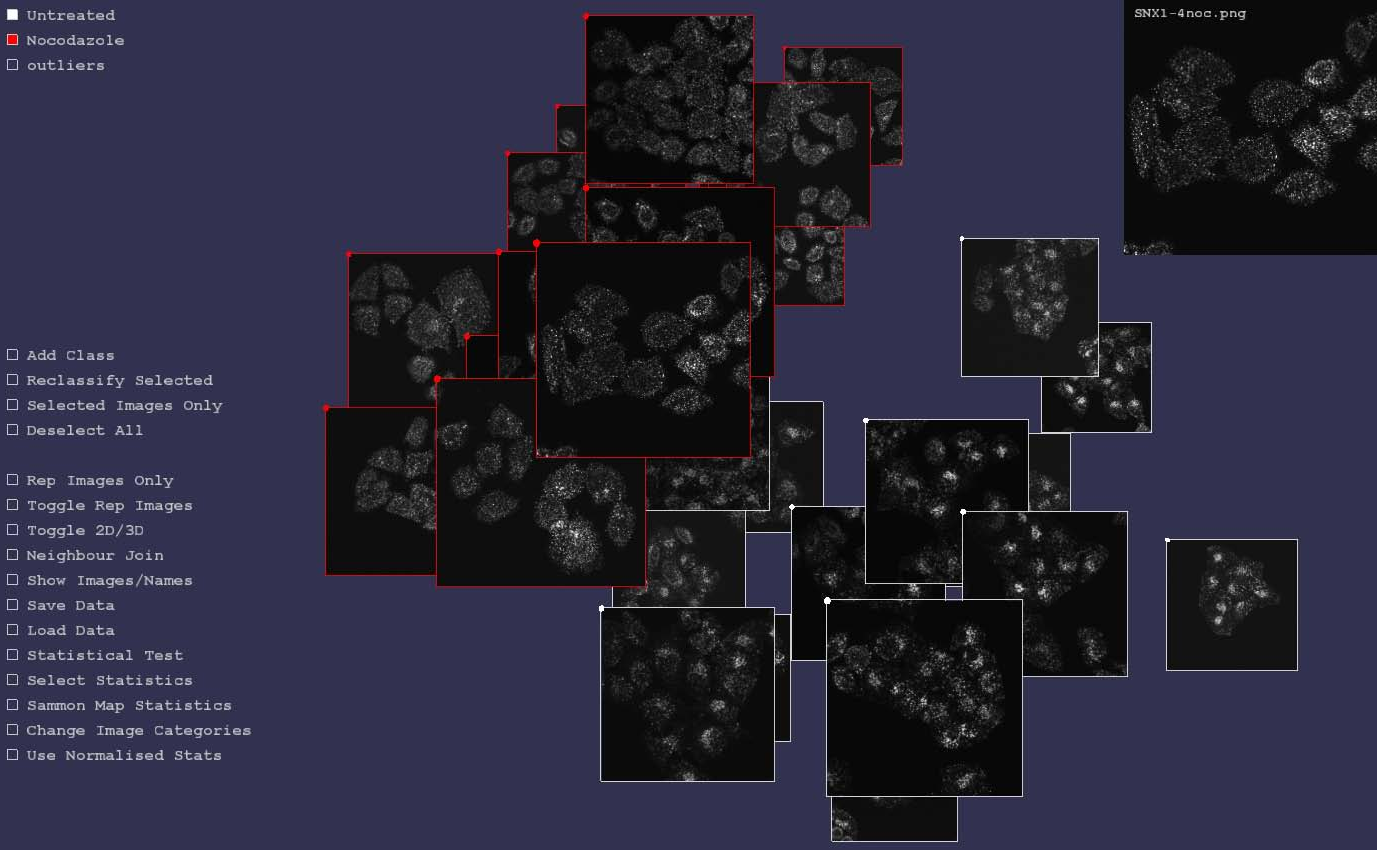
**Distinguishing untreated/treated protein localisation**. Two sets of images are visualised in iCluster: one for which the protein SNX1 has been fluorescently tagged and imaged (white borders); and one for which SNX1 has similarly been imaged in cells treated with nocodazole (red borders). For each image, TAS are generated and mapped into 3 dimensions. The visual difference between nocodazole treated and control images is quite subtle with the control images exhibiting slightly more peri-nuclear clustering than the nocodazole treated case. However, the above result of statistic generation and mapping clearly shows that the treated and control images have been distinguished. Each image is shown connected to its nearest neighbour in the 3D space. The top right corner shows the current mouse selected image in more detail.

To test the ability of the methodology to detect small changes in imaging, two image sets were created: the endosomal protein SNX1 was fluorescently labelled using SNX1-specific antibodies and imaged in cells treated with nocodazole (16 images) or the carrier control (17 images). See Image Set B in Methods for experimental details. SNX1 is an endosomal protein (19), and nocodazole disrupts the microtubule network that is involved in endosomal transport and subcellular distribution (20). Hence untreated cells present a more clustered concentration in the peri-nuclear region, while nocodazole treated cells exhibit a more even distribution of endosomes throughout the cell. Testing the SNX1 imaging against SNX1+nocodazole, gave a p-value for the null hypothesis of 0.000, and hence the relatively subtle difference in images was readily detected (Figure 4). Hence the statistical testing regime outlined shows a high degree of discrimination.

To test if the methodology might be sensitive to detecting random variability, repeat experiments were performed. Using the procedure outlined in Methods for Image Set C, cells expressing fluorescently labelled LAMP1 were prepared. One set of cells was imaged on one day, and another on the consecutive day. The cells were divided into three separate populations corresponding to wells: two wells from day 1 and one well from day two. The images from the 3 wells were then compared pair-wise by randomly selecting 12 images of each well and generating a p-value for the null hypothesis of no change. Repeating the random selection 100 times then gave an average p-value for each pair of wells. The wells imaged on the same day gave an average p-value of 0.392, while comparing wells imaged on distinct days gave p-values of 0.316 and 0.300. While the p-values are lower when comparing wells from distinct days, they would not give cause to reject the null hypothesis. Hence, with careful control of experimental conditions the chance of detecting change where there is none is reduced.

It should be strongly emphasised that as image statistics become more sensitive there is a real danger of detecting differences in the imaging conditions or the hardware setup rather than real changes in localisation. Hence the ideal experiment is to compare image sets for which the classes to be compared are imaged at the same time on a single plate in distinct wells with identical technical specifications.

### Rejection of the null hypothesis

One potential problem with randomised permutation methods is rejection of the null hypothesis may occur at too high a rate [16]. To test the null hypothesis rejection rate, randomly chosen subsets of 15 images of the endoplasmic reticulum from image set A were selected. For two such (disjoint) sets, a p-value for the null hypothesis was calculated. This was repeated 10,000 times, to give 10,000 p-values. Of the 10,000 p-values, the null hypothesis was rejected (p > 0.95) 510 times, which is close to the expected number of 500. Further, binning the p-values into intervals of length 0.05, each bin contained 500 +/- 44, showing that the distribution of p-values is relatively flat. Hence it can be concluded that rejection of the null hypothesis is occurring at approximately the correct rate.

### Comparison to previous tests

As described in Methods, in [17] several statistical tests for difference were compared, and it was shown the most sensitive for subcellular image statistics was the 3-neighbour test [18]. It was shown that using around 40 images of individual cells of each subcellular localisation and applying this test, the null hypothesis could be rejected at a rate of 90%.

Here we compare the centroid distance test and the 3-neighbour test using TAS calculated for subsets of the plasma membrane and actin cytoskeleton image sets. Random subsets of n images of each class, n from 5 to 15, were selected and a p-value for both tests calculated. For each n, 100 tests were completed, and the averages are shown in Table 3. It can be seen that the p-values for the centroid distance test are up to a factor of 2 lower than the p-values for the 3-neighbour test, though for larger n both tests give small p-values for the null hypothesis. For each n, the table also shows how many of the 100 tests reported a p-value greater than 0.05, ie a relatively large p-value for the null hypothesis. For all cases the centroid distance test gave a lower number of relatively high p-values for the null hypothesis. Hence we conclude that the centroid distance test is more sensitive for detecting difference in subcellular imaging.

**Table 3:** Comparing centroid distance and 3-neighbour tests

n	**Average** **p-value**		**% with p > 0.05**	
	
	Distance	3-nbrs	Distance	3-nbrs
5	0.0882	0.1969	62	73

6	0.0683	0.0115	47	54

7	0.0431	0.0988	30	45

8	0.0274	0.0502	21	34

9	0.0204	0.0311	10	22

10	0.0126	0.0179	4	7

11	0.0121	0.0157	5	8

12	0.0060	0.0079	2	4

13	0.0050	0.0027	0	0

14	0.0038	0.0018	0	0

15	0.0026	0,0010	0	1

For a given number *n *of images, random subsets of *n *plasma membrane and *n *actin cytoskeleton images were selected and a p-value calculated for the centroid distance statistic and the 3-neighbours statistic. For a given *n*, the average of 100 p-values is shown. For each n, the percentage of the 100 tests that reported p > 0.05 for the null hypothesis is also shown.

### Computational expense

To load the 500 images of Image Set A into iCluster and calculate TAS took 70 seconds. To calculate the spatial layout of the images (Sammon map) took approximately 5 minutes. It should be noted that while the calculation of TAS is essentially linear in the number of images, the calculation of the Sammon map it not. Hence calculation of spatial layout for 100 images may only take 2–5 seconds. Calculation of p-values (1000 repeats) for moderate size image pairs set (50 images each) is essentially instantaneous from the user's point of view. Hence for moderate size (less than 100) image sets, the images can be loaded, statistics and layout calculated, and p-values found in a few 10's of seconds.

Testing was conducted on an Intel Core Duo 2 T5600 notebook with nVidia GeForce Go 7900 GS graphic card under the Fedora Core 8 Linux operating system.

## Conclusion

The intention here has been to provide a new statistical test and a protocol for detecting difference in subcellar fluorescent microscopy imaging. It has been shown that the major subcellular localisations may readily be distinguished with as few as 12 images from high throughput microscopes, and that subtle shifts in localisation such as endosomal redistribution can be automatically detected. It has also been shown that outlier images may easily be detected from large image sets by visual inspection, and that their removal can significantly improve confidence in null hypothesis testing. In some experiments it may be the outliers that are the most interesting images in that an unusually high number of cells are not expressing the protein in the expected manner. Further, the statistical testing framework utilising permutation testing has been rigorously evaluated to show that the p-values generated reject the null hypothesis at the expected rate and that the sensitivity is higher than previous approaches.

A significant advantage of the methodology outlined is in speed of computation. Previous comparison of computing time for TAS and the commonly used Haralick measure showed TAS to be 30 times faster to calculate [5]. Few image statistics are as computationally simple as TAS. Hence for high throughput screening applications, an implementation of TAS with the centroid difference test could detect those experiments in which treatment has changed protein localisation in days rather than months of computational time. It is also worth noting that as a simple, fast and sensitive test, the centroid distance test could readily be implemented in high throughput screening pipelines without utilising iCluster. Indeed, we plan to apply TAS and the centroid difference test for screening applications in the near future. Another advantage over previous approaches is that it can operate with or without cell selection, hence reducing computational expense and variability of results due to differing levels of success in the selection procedure.

It should be emphasised that care was taken to avoid human intervention in the preparation of the image sets, and to use microscopes and microscope settings commonly used for high throughput imaging. As far as we are aware this is the first study on testing for difference in subcellular imaging that utilises high throughput images that have not been selected by human intervention in any way. This gives strong confidence that the results obtained will be applicable and reproducible in "real" applications.

A feature of iCluster is that it may equally well operate with user supplied statistics. A simple text file format outlined in the user manual may be used to describe each image and a set of statistics associated with it. iCluster will then calculate spatial layout and do statistical testing just as has been shown here for TAS. Similarly, iCluster can operate with user supplied statistics but without images being supplied, in which case each data points is represented as a simple sphere. Hence the methodology is not limited to subcellular localisation imaging and could be applied to any data or image set for which the researcher has generated some form of statistics.

As such we foresee many applications of iCluster to visual data exploration. As an example, in collaboration with other members of the Institute for Molecular Bioscience, iCluster has been used to explore data from tri-localisation experiments in cells (B. Woodcroft, L. Hammond, J. Stow, N. Hamilton: Automated organelle-based colocalisation in whole cell imaging, submitted). Each data point corresponded to an endosome from a cell, with 7 numbers describing the degree of overlap of each of 3 fluorescent markers on that endosome. With some 875 endosomes in one data set, iCluster was utilised to map the set of 7 dimensional vectors associated with the endosomes into 2 dimensions. In this representation the data naturally fell into a triangle, with each vertex of the triangle corresponding to one of the three markers used in the experiment, and points within the triangle corresponding to varying degrees of colocalisation of the proteins. In this way it was then possible to view and make sense of the whole data set and the diversity of the (co)localisations of the proteins marked on each of the endosomes in a way that was not possible by viewing a spreadsheet of the data. As bio-data sets become increasingly larger there is an urgent need for tools to explore and make sense of them, and we believe that iCluster will be invaluable in visual data exploration.

## Methods

### Image data sets

#### Image Set A

**Figure 5:**
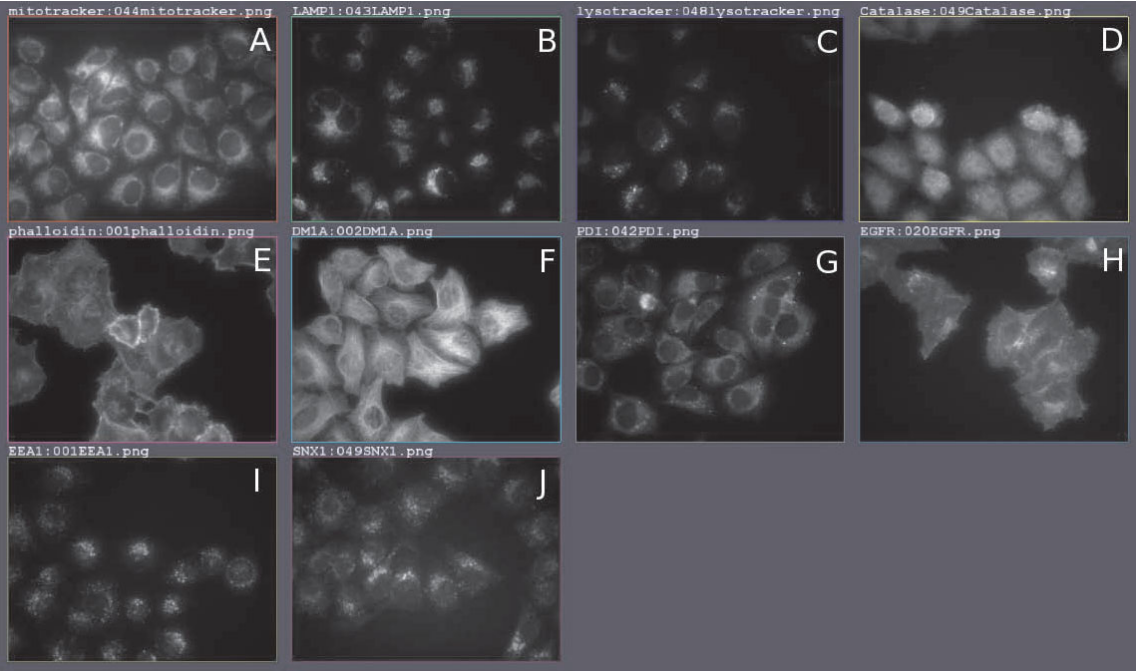
**Representative images for the 10 subcellular localisations of Image Set A**. A natural choice for a representative image of an image set is to choose the image that has statistics closest, in the Euclidean sense, to the centroid of the image statistics for that set [12,13]. The above shows representative images found and visualised in iCluster using threshold adjacency statistics in this way. Subcellular localisations shown are (A) Mitochondria (B) Late endosome/lysosome (C) Lysosome (D) Peroxisome (E) Actin (F) Microtubules (G) Endoplasmic reticulum (H) Plasma membrane (I) Early endosome (J) Endosome.

An image set comprising of 10 subcellular localizations was obtained, representing 10 distinct subcellular organelles. Each organelle image set consists of 512 localization images, equating to a total of 5120 localization images overall. From these images, 50 images per localisation were randomly chosen for the purposes of this paper. HeLa (cervical cancer) cells were seeded onto a 96 well plate, fixed, and then labelled using fluorescent antibodies against endogenous proteins or structures. Labels were chosen as known markers of the subcellular localisations: peroxisome (catalase), microtubules (DMA1, alpha-tubulin), early endosome (EEA1), plasma membrane (EGFR), late endosome/lysosome (LAMP1), lysosome (lysotracker), mitochondria (mitotracker), endoplasmic reticulum (PDI), actin cytoskeleton (phalloidin), and endosome (SNX1). The image capture process was automated in a high-throughput manner, utilizing a 40× dry lens objective, autofocused with a fixed exposure time on the BD Pathway 855 to image the cells without human intervention. Note that image capture was fully automated and care was taken not to adjust microscope settings or select the images in any way. The images are 8-bit greyscale, 672 × 512 pixels, each containing up to 20 cells. Automatically selected representative images for each class are shown in Figure 5.

#### Image Set B

A nocodazole treated versus control image collection was generated by imaging endogenous sorting nexin 1 (SNX1) in A-431 (human epithelial carcinoma) cells treated with 10 μM nocodazole (Sigma Aldrich) or equivalent concentrations of the carrier (dimethyl sulfoxide) for 30 min (nocodazole treatement disrupts the microtubule network of the cell (20)). Endogenous SNX1 was detected with a monoclonal antibody raised against the first 108 amino acids of human SNX1 (BD Biosciences). Confocal Z-stacks (0.7 μm) of the entire volume of the monolayers were captured on a Zeiss LSM 510 confocal scanning microscope using a 63× oil objective. Maximum projections were generated using the LSM software (Zeiss). In total there were 17 treated and 16 untreated images captured at 512 × 512 resolution.

#### Image Set C

Repeat experiments of the LAMP1 marker were performed in the manner described for Image Set A. Imaging occurred on two distinct days. The image set consists of 64 images each from two distinct wells imaged on day 1, and a further 64 images from a single well captured on day 2.

Image sets are available for download from the LOCATE database home page [19].

### Image Statistics

A wide variety of classes of image statistics have been tested for their capacity to distinguish images of sub-cellular localization, primarily for use in image classification. Conrad et al. [4] tested 448 different image features and applied a variety of feature reduction and machine learning methods. Of those tested, Haralick texture measures [20], sometimes known as co-occurrence measures, were found to give the best performance. Subsequently, our group introduced threshold adjacency statistics (TAS), and found that these statistics in combination with machine learning methods could provide comparable classification accuracy (up to 95%) to the Haralick measures while being at least an order of magnitude faster to calculate [5]. Further, TAS may be used with or without selecting individual cells from an image and do not require a separate image to identify the nuclear region. Hence for reasons of speed and simplicity, TAS are utilised here for visual and statistical testing of difference. Each image is then associated with a vector of 27 real numbers calculated from TAS.

Briefly, TAS are generated by first applying an adaptive threshold range to the image to create a binary image. Nine statistics are then calculated from the binary image. For each white pixel, the number of adjacent white pixels is counted. The first threshold statistic is then the number of white pixels with no white neighbours; the second is the number with one white neighbour, and so forth up to the maximum of eight. The nine statistics are normalised by dividing each by the total number of white pixels in the threshold image. Two other sets of threshold adjacency statistics are also calculated as above, using two other threshold ranges, giving in total 27 statistics. Note that in order that each statistic be given equal weighting in the subsequent calculations, each is normalised by subtracting the mean for that statistic for an image set and dividing by the standard deviation. Details may be found in [5].

### Statistical testing for difference

The Hotelling T^2 ^test [21], a multivariate form of the student t-test, has previously been applied to the problem of statistical differentiation of subcellular imaging [22]. However, there are a number of difficulties with this approach. The test assumes that each of the statistics has a normal distribution, which is often not the case for statistics generated from subcellular imaging. Further, the test requires there to be more images in each class being tested than the number of statistics generated for each image. With anywhere from 27 [5] to 57 [7] statistics generated for subcellular imaging, this can severely limit the application of the test. In [17], it was shown that using around 40 images of cells each of the major subcellular localisations could be differentiated using the Friedman-Rafsky test that utilises minimal spanning trees, or by k-nearest neighbour testing. Briefly, in the k nearest neighbour test, the nearest neighbors of each of the data points are examined, the nearest neighbors of a data point being those data points that are closest to the point as measured by the Euclidean distance. For a given data point, the number of the k closest points (k nearest neighbors) to that point that are of the same class as the point is recorded. The test statistic is then the total number of k nearest neighbors of elements of a set that are also in that set [18].

Both approaches use statistics on the classes of the neighbours of each image, and whether those neighbours are of the same class. Hence these tests are to some degree measuring the disjointness of the statistics of the image sets being compared.

Towards detecting shifts in the statistical centres of image sets rather than the discreteness of clusters, the approach taken here is via a centroid distance test employing permutation testing [16]. To test for difference between two image sets I_1 _and I_2_, 27 TAS are generated for each image in the sets. The mean statistics vectors μ(I_1_) and μ(I_2_) are then calculated for each, together with the Euclidean distance d(μ(I_1_), μ(I_2_)). The null hypothesis is that the image statistics of I_1 _and I_2 _are drawn from the same distribution, more specifically that the population means are the same : μ_I1 _= μ_I2_. To test this, the observations of I_1 _and I_2 _are randomly permuted to give sets R_1 _and R_2 _which have the same sizes as I_1 _and I_2_, respectively, but may have statistics vectors from either. The distance d(μ(R_1_), μ(R_2_)) is then calculated. Repeating 1000 times, the fraction of the repeats for which d(μ(R_1_), μ(R_2_)) > d(μ(I_1_), μ(I_2_)) then gives a p-value for the null hypothesis. For image sets for which there is a detectable difference, it would be expected that the mean vectors would be more separated, on average, than the randomisations, hence giving a small number of repeats for which d(μ(R_1_), μ(R_2_)) > d(μ(I_1_), μ(I_2_)).

## Abbreviations

TAS: Threshold adjacency statistics; HeLa cells: Cervical cancer cells named after their donor, Henrietta Lacks; A-431 cells: Human epithelial carcinoma cells.

## Authors' contributions

NH designed and tested the centroid distance test, the iCluster work flow and drafted the manuscript. JW & MK created the experimental image sets for the study and contributed to the design of the study. RT participated in the design of the study and coordination and helped draft the manuscript. All authors read and approved the final manuscript.

## Supplementary Material

Additional file 1 **An example of using iCluster.** Initially, 50 mitochondria images (mitotracker) and 50 plasma membrane images (EGFR) are shown randomly placed having been loaded into iCluster and statistics calculated. 'Sammon Map Statistics' is then selected and the images move around as a spatial layout is found that reflects the distances between the statistics vectors for the images. The user then rotates the image set, and 3 outlier images are observed, selected (red tint), and then show in more detail in a 2D representation. All three appear to contain artefacts. The view then switches back to the 3D view, a new class 'outlier' is added to the class list, the selected images are reclassified to this class (green borders), and then removed from view by deselecting their class button. Representatives for each class of the remaining images are then shown side by side in a 2D view. The view then changes back to the 3D view, and 'Statistical Test' selected. The images to compare and the number of repeats to calculate a p-value for the null hypothesis (no difference) are then selected. Finally, the returned p-value of 0.000 is displayed, showing that the visual assessment of difference is confirmed statistically. Click here for file
